# Clinical Assessment of Medical Students in the Emergency Department, a National Consensus Conference

**DOI:** 10.5811/westjem.2016.11.32686

**Published:** 2016-11-23

**Authors:** Katherine M. Hiller, Douglas Franzen, Luan Lawson, David Manthey, Jonathan Fisher, Marianne Haughey, Matthew Tews, Nicole Dubosh, Joseph House, Arleigh Trainor, David Wald, Julianna Jung

**Affiliations:** * University of Arizona, Department of Emergency Medicine, Tucson, Arizona; † University of Washington, Department of Medicine, Division of Emergency Medicine, Seattle, Washington; ‡ East Carolina University, Department of Emergency Medicine, Greenville, North Carolina; § Wake Forest University, Department of Emergency Medicine, Winston-Salem, North Carolina; ¶ St. Barnabas Medical Center, Department of Emergency Medicine, Bronx, New York; || Medical College of Wisconsin, Department of Emergency Medicine, Milwaukee, Wisconsin; # Harvard University, Department of Emergency Medicine, Cambridge, Massachusetts; ** University of Michigan, Department of Emergency Medicine, Ann Arbor, Michigan; †† University of South Dakota, Department of Emergency Medicine, Vermillion, South Dakota; ‡‡ Lewis Katz School of Medicine, Philadelphia, Pennsylvania; §§ Johns Hopkins University, Department of Emergency Medicine, Baltimore, Maryland

## BACKGROUND

The clinical assessment of medical students in the emergency department (ED) is a highly variable process in which clerkship directors (CD) create and use institution-specific tools, many with unproven reliability or validity, to assess students of differing experience and from different institutions. 1,2

## OBJECTIVES

Standardization of assessment practices and tools of assessment could enhance grading, improve the reliability and validity of information on the standardized letter of evaluation (SLOE) for program directors, and most importantly, provide consistent, valid and reliable formative feedback for students.

## DESIGN

A consensus conference on end-of-shift assessment of medical students in the ED was held in the Clerkship Directors in Emergency Medicine (CDEM) track of the Council of Emergency Medicine Residency Directors (CORD) Academic Assembly in Nashville, TN, in March 2016. Themes surrounding the practice of end-of-shift assessment of medical students were derived from small-group discussions among the executive committee and refined at a large-group planning meeting at the 2015 CORD Academic Assembly (Table).

In May 2015, theme leaders were identified and tasked with recruiting relevant stakeholders to their respective small groups, synthesizing the background literature and articulating key issues surrounding their theme. Simultaneously, the executive committee derived “building blocks” of assessment from foundational source materials. 1, 3–9 Each contained the following: name, background and definition, benefits/drawbacks/alternatives to use in the clinical setting, areas of overlap with other domains of assessment, examples of how an assessment of this domain would appear on an assessment form in three formats (narrative, dichotomous, and an anchored ratings scale), and references.

On Day 1 of the conference, participants were divided into small groups. Each theme leader met with each small group providing background and guiding further discussion. Pre-determined questions with discrete responses were asked within each small group. During the second morning of the conference, the “building blocks” were discussed. Participants voted using an electronic audience response system (www.polleverywhere.com).

## IMPACT/EFFECTIVENESS

Sixty people participated on Day 1 and 70 participated on Day 2 of the conference. Participants agreed on 63.4% of the theme questions and 87.5% of the domains of assessment. The group felt that both norm- and criterion-based assessment should be incorporated, EM faculty and senior residents should be allowed to complete the form, the unit of observation should be a single shift, and that 6–10 shifts would be adequate to accurately assess a student. Medical students (MS3) and (MS4) should be assessed using the same tools, but grading should differ. Learners with varying experience within a year present a challenge; however, this is not prohibitive to using a common form or grading rubric. Clinical assessment data should be translated into a grade and onto the SLOE. Of 16 domains of assessment presented, nine were included, five omitted, and two did not reach consensus. All domains should be assessed via rating scale except professionalism, for which a combined narrative/dichotomous approach was preferred.

Based on the variability of assessment forms currently in use, we anticipated a large range of opinion on the topics presented. Instead, we were surprised by the strength of consensus on most topics.

Limitations to this process include that only approximately half of the CDEM Academy membership was present, despite extensive advertisement about the conference. Additionally, voting may have been affected by the order in which the building blocks of assessment were presented. Participants may have been more apt to comment later once they had a better understanding and more familiarity with how the materials were presented and referenced. We attempted to mitigate this effect by providing the materials to participants beforehand and providing preparatory background material in discussion groups. Finally, participants were able to change their vote while group discussion occurred. Large-group discussion did sway votes; however, we feel this culminated in a better representation of the group’s actual opinions. Discussion helped guide the decision in real time, and allowed minority opinions to be heard and considered.

This conference was a critical first step in the development of national guidelines and a standardized clinical assessment tool in EM. The education and discussion that the conference provided elevated the level of conversation around assessment in our specialty. The creation of a reliable and valid assessment tool will provide a critical method for measuring outcomes in educational innovations and research in the future.

Please see Appendix for CDEM Consensus Conference on End-of-shift Assessment of Medical Students: Executive Summary.

## Supplementary Information



## Figures and Tables

**Table:** Themes of assessment discussed at the CDEM national end-of-shift consensus conference.

Themes
Criterion vs norm-referenced assessment
Learners at different levels of learning
Translation of assessment data into other products
Utilization of clinical assessment tools
Ensuring post-implementation validation/research

*CDEM*, Clerkship Directors in Emergency Medicine.
