# Supplementary material for: Clinical Assessment of Medical Students in the Emergency Department, a National Consensus Conference
Source: West J Emerg Med. 2016 Nov 23;18(1):82–3. doi: 10.5811/westjem.2016.11.32686 (PMC5226769; doi:10.5811/westjem.2016.11.32686)
Supplement: Supplementary file 1 [file wjem-18-82-s001.pdf]

The CDEM Consensus Conference on End-of-shift Assessment of Medical Students: Executive Summary

Katherine Hiller, MD, MPH*
Doug Franzen, MD, M.Ed†
Luan Lawson, MD, MAEd‡
Julianna Jung, MD§

**University of Arizona, Department of Emergency Medicine, Tucson, Arizona
†University of Washington, Department of Medicine, Division of Emergency Medicine, Seattle, Washington
‡East Carolina University, Department of Emergency Medicine, Greenville, North Carolina
§Johns Hopkins University, Department of Emergency Medicine, Baltimore, Maryland

Section Editor: Douglas S. Ander, MD
Submission history: Submitted June 15, 2016; Revision received September 30, 2016; Accepted October 27, 2016
Electronically published [date]
Full text available through open access at http://escholarship.org/uc/uciem_westjem
DOI: 10.5811/westjem.2016.10.31266

The need to incorporate best practices and to standardize the process of clinical evaluation in undergraduate emergency medicine led to the concept of developing a set of national guidelines and a common form via a consensus conference. The goal of the first half of the conference was to derive consensus surrounding best practices of clinical assessment, and the goal of the second half was to create a national end-of-shift assessment form. Specific themes surrounding the practice of end-of-shift assessment of medical students were determined in preconference planning sessions. Using foundational source material, "building blocks" of assessment were developed. Participants were given all conference materials to review prior to the conference. Small group discussions on the predetermined themes of assessment were conducted on the first half day. The participants discussed and voted on the "building blocks" during the second half day using an electronic polling system, Poll Everywhere®. A supermajority was considered definitive, and consensus was reached on a surprisingly high proportion of issues and "building blocks." Ongoing post-conference work includes a modified Delphi process to reconcile disputed issues, development of the national form, and research on implementation, validation and reliability of the form. [West J Emerg Med. 20XX;XX(X)XX-XX.]

Over the past two decades, there has been an increased interest in clinical assessment methods in undergraduate and graduate medical education. In 1999, the Accreditation Council for Graduate Medical Education (ACGME) described the Core Competencies.[1] The ACGME continued the Outcomes Project with the Next Accreditation System in 2012, introducing "Milestones."[2] Milestones have been defined for residents as well as medical students in emergency medicine (EM).[3,4] Finally, in 2014 the Association of American Medical Colleges developed a list of Entrustable Professional Activities (EPA) that medical students are expected to master prior to beginning internship.[5,6]

During this same time frame, national task forces developed EM curricula for both third- and fourth-year medical students, and two national standardized EM examinations; and the Standardized Letter of Evaluation (SLOE) has been updated and revised periodically.[7-10] Despite these advances, clinical assessment of students has remained largely institutionally driven, reliant on individual clerkship directors creating their own assessment tools and practices.[11] Standardization of both the process of clinical assessment, as well as the tools used, are critical next steps in improving reliability and validity of assessment within and across institutions.

THE CONCEPT OF A CONSENSUS CONFERENCE

At the 2013 Clerkship Directors in Emergency Medicine (CDEM) conference, a group of members met to discuss issues and challenges in assessment, initiating a survey on the current state of clinical assessment in EM.[12,13] Respondents expressed interest in the development of national assessment guidelines and a standardized assessment tool. Due to extreme variability in the current state of clinical assessment, it was necessary

to design a process to gather input from all stakeholders in the clinical assessment process, including students, clerkship directors, residency directors, and deans. The format of a consensus conference has been successfully used to synthesize disparate practices involving multiple stakeholders.[14] The Council of Emergency Medicine Residency Directors (CORD) Academic Assembly was chosen for the conference, as it unites educators in both undergraduate medical education and graduate medical education and is focused entirely on education-related topics. The relevant planning committees were approached two years prior to the proposed conference, and they committed to providing space and time.

Setting the Task

Once the format, time and venue were identified, the executive committee consulted content and process experts. Standardizing practices surrounding assessment was agreed to be as important as developing a common assessment tool. To this end, a two-part conference was conceived with the first half focused on best practices in assessment, and the second half development of a national end-of-shift assessment tool.

Themes underpinning end-of-shift assessment were derived from small group discussions among the executive committee, and refined at a large-group planning meeting at the 2015 CORD Academic Assembly. They were the following:

1. Criterion- vs norm-referenced assessment
2. Assessing learners at different levels of learning
3. Translation of end of shift assessment data into other products (SLOEs, final clerkship grades, medical school performance evaluations)
4. Implementation and use of assessment tools
5. Ensuring post-implementation evaluation and validity.

The executive committee identified theme leaders and invited them to participate in May 2015. To capitalize on the opportunity for mentorship and to engage both experienced and new clerkship directors, "senior" theme leaders were paired with "junior" theme leaders whenever possible. Each theme leader recruited relevant stakeholders to their group, including clerkship directors, residency program leaders, SLOE taskforce members, and non-physician education experts. All groups participated in regular conference calls to ensure cross-communication and global coordination of key tasks. An additional in-person meeting took place at the 2015 American College of Emergency Physicians Academic Assembly.

Conference Preparation

Part 1: Identifying Best Practices

Literature searches were initiated within each theme. Nominal group technique was used to articulate major sub-concepts within each theme, possible resolutions to disparate opinions, and the educational landscape that put these decisions in context. Theme leaders participated in other themes' discussions to assure complete and non-duplicative efforts.

The executive committee summarized the current state of assessment by synthesizing findings from cognitive psychology and education literature, reporting the results of the aforementioned national survey, and conducting a frequency analysis of domains assessed on 79 assessment tools currently in use. Finally, cognitive psychology and education literature were consulted for best practices, when appropriate.

Part 2: Building a National Clinical Assessment Tool

The executive committee used foundational source material (ACGME Milestones, EPAs, etc.) as well as a collection of current tools to develop "building blocks" for discussion by conference participants.[1,3,8,10,13] Each building block represented a domain of assessment and contained the following elements: name, definition, benefits/drawbacks/alternatives to inclusion on the final tool, any overlap with other domains of assessment, and examples of how it may appear on an assessment form in three formats (narrative, dichotomous, and an anchored rating scale).

The Conference

The conference was publicized to clerkship directors, residency directors, deans, and non-physician educators. Participants registered for the conference concurrently with the CORD Academic Assembly. One week prior to the conference, reference materials were distributed electronically to registered attendees.

The conference was divided into two half-day events occurring on separate days. This format allowed the executive committee and theme leaders to debrief and analyze results between sessions and gave participants time to "digest" the material from the first day, and return on day 2 with renewed energy and focus.

The first day began with brief summary lectures on the current state of clinical assessment in the emergency department (ED), an introduction to the themes for discussion, and the ground rules for the consensus process itself. Conference participants were divided into small groups for in-depth discussion of issues and consensus building. Theme leaders rotated rooms to lead discussions on their content for each small group. Attendance for each breakout group was recorded, and the input of each participant was sought. Scribes documented the discussion. Using paper ballots, at the end of each session, participants voted on three to four pre-determined key questions with categorical or binary responses.

Theme leaders debriefed after the first session, analyzing the data and summarizing major findings. The second session of the conference began with a recap and synthesis of the findings from the first day followed by presentation of the building blocks. Source materials and the building block documents distributed prior to the conference were available

for participants to view on paper as well as being projected in real time.[2-5,7,8,10]

Voting was recorded via the Poll Everywhere® electronic audience response system (www.polleverywhere.com). For each assessment building block, participants voted on the following:

1. Whether the domain should be included on a national clinical assessment tool
2. If yes, whether the domain would best be assessed via a narrative response, a dichotomous assessment or a rating scale, and
3. How the assessment for that domain could/should be modified (qualitative free response)

The first two questions were simple yes/no and multiple choice. A 66% supermajority (decided a priori) was considered definitive. If a supermajority was not reached, a short discussion period was held, and voting re-opened. The last question was answerable via qualitative text response. In addition to electronic responses, a scribe recorded discussion within the large group.

Post-conference Work

Following the conference, the results were disseminated electronically to participants and presented at the national Society for Academic Emergency Medicine conference in April 2016. A modified Delphi process is currently underway to refine and finalize the work of the conference. All conference participants and other EM educators were invited to participate in the Delphi group, with the goals of answering unresolved consensus questions and finalizing the national assessment tool

CONCLUSION

This consensus conference successfully initiated the development of a national tool and best practice guidelines for clinical assessment of medical students in the ED. Implementation required engagement from a large number of educators to be engaged, and also the national organization hosting the meeting.

To maximize productivity, it was essential to narrow the focus to specific questions, and to frame the discussion prior to the conference itself. While the executive committee members were very familiar with the background concepts on which decisions rested, it was necessary to spend time ensuring that theme leaders understood the framework, and that conference attendees were educated about the underpinnings of the discussion. This required a significant amount of pre-conference work.

Finally, the *a priori* defined level of consensus was not reached on some issues, as anticipated. Post-conference work is required to resolve differences and synthesize agreeable compromises.

Address for Correspondence: Katherine Hiller, MD, MPH, University of Arizona, Department of Emergency Medicine, 1501 N Campbell Ave., Tucson, AZ 85724. Email: khiller@aemrc.arizona.edu.

Conflicts of Interest: By the *West*JEM article submission agreement, all authors are required to disclose all affiliations, funding sources and financial or management relationships that could be perceived as potential sources of bias. The authors disclosed none.

Copyright: © 2016 Hiller et al. This is an open access article distributed in accordance with the terms of the Creative Commons Attribution (CC BY 4.0) License. See: http://creativecommons.org/licenses/by/4.0/

REFERENCES

4. Lurie SJ, Mooney CJ, Lyness JM. Measurement of the general competencies of the accreditation council for graduate medical education: a systematic review. *Acad Med.* 2009;84(3):301-309.
5. Nasca TJ, Philibert I, Brigham T, et al. The next GME accreditation system--rationale and benefits. *N Engl J Med.* 2012;366(11):1051-1056.
6. The emergency medicine milestones project. Available at: http://www.acgme.org/acgmeweb/portals/0/pdfs/milestones/internalmedicinemilestones.pdf. Accessed April 5, 2016, 2016.
7. Santen SA, Peterson WJ, Khandelwal S, et al. Medical student milestones in emergency medicine. *Acad Emerg Med.* 2014;21(8):905-911.
8. AAMC. Core Entrustable Professional Activities for Entering Residency--Curriculum Developers Guide. Available at: https://members.aamc.org/eweb/upload/core%20EPA%20Curriculum%20Dev%20Guide.pdf. Accessed Feb 11, 2016.
9. AAMC. Core entrustable professional activities for entering residency: Faculty and learners' guide. Available at: http://members.aamc.org/eweb/upload/Core%20EPA%20Faculty%20and%20Learner%20Guide.pdf. Accessed Nov 15, 2015.
10. Manthey DE, Ander DS, Gordon DC, et al. Emergency medicine clerkship curriculum: an update and revision. *Acad Emerg Med.* 2010;17(6):638-643.
11. Tews MC, Ditz Wyte CM, Coltman M, et al. Implementing a Third-Year Emergency Medicine Medical Student Curriculum. *J Emerg Med.* Mar 27 2015.
12. Senecal EL, Heitz C, Beeson MS. Creation and implementation of a national emergency medicine fourth-year student examination. *J Emerg Med.* 2013;45(6):924-934.
13. Love JN, Smith J, Weizberg M, et al. Council of Emergency Medicine Residency Directors' standardized letter of recommendation: the program director's perspective. *Acad Emerg Med.* 2014;21(6):680-687.
14. Hiller K, Lawson L, Franzen D., et al . End-of-shift Emergency Medicine Medical Student Clinical Evaluation Forms: A Taxonomy. *Acad Emerg Med.* 2015;22(S1):S196-197.

15. Lawson L, Jung J, Franzen D, et al. Clinical Assessment of Medical Students in Emergency Medicine Clerkships: A Survey of Current Practice. *J Emerg Med.* Sep 7 2016.
16. Lawson L. Characterization of Utilization of Clinical Assessment Tools in Emergency Medicine Medical Student Education. In: Hiller K, ed. San Diego, CA: Society for Academic Emergency Medicine Annual Meeting; 2015.
17. Waggoner J, Carline JD, Durning SJ. Is There a Consensus on Consensus Methodology? Descriptions and Recommendations for Future Consensus Research. *Acad Med.* 2016;91(5):663-668.
